# In Situ Formed Protective Barrier Enabled by Sulfur@Titanium Carbide (MXene) Ink for Achieving High‐Capacity, Long Lifetime Li‐S Batteries

**DOI:** 10.1002/advs.201800502

**Published:** 2018-07-04

**Authors:** Huan Tang, Wenlong Li, Limei Pan, Conor P. Cullen, Yu Liu, Amir Pakdel, Donghui Long, Jian Yang, Niall McEvoy, Georg S. Duesberg, Valeria Nicolosi, Chuanfang (John) Zhang

**Affiliations:** ^1^ College of Materials Science and Engineering, and Jiangsu Collaborative Innovation Center for Advanced Inorganic Function Composites Nanjing Tech University Nanjing 210009 P. R. China; ^2^ Centre for Research on Adaptive Nanostructures and Nanodevices (CRANN) & Advanced Materials Bio‐Engineering Research Centre (AMBER) School of Chemistry Trinity College Dublin Dublin 2 Ireland; ^3^ School of Chemical Engineering East China University of Science and Technology Shanghai 200237 P. R. China; ^4^ Institute of Physics EIT 2 Faculty of Electrical Engineering and Information Technology Universität der Bundeswehr Werner‐Heisenberg‐Weg 39 München Neubiberg 85577 Germany

**Keywords:** Li‐S batteries, MXene, polysulfide shuttles, protective barriers, sulfate complexes

## Abstract

Sulfur (S) is an attractive cathode material with advantages including high theoretical capacity and low cost. However, issues such as the lithium polysulfide shuttle effect and its insulating properties greatly limit the future applications of lithium‐sulfur (Li‐S) batteries. Here, a viscous aqueous ink with nanoscale S uniformly decorated on the polar, metallically conductive titanium carbide MXene nanosheets (S@Ti_3_C_2_T*_x_*) is reported to address these issues. Importantly, it is observed that the conductive Ti_3_C_2_T*_x_* mediator efficiently chemisorbs the soluble polysulfides and converts them into thiosulfate/sulfate. The in situ formed sulfate complex layer acts as a thick protective barrier, which significantly retards the shuttling of polysulfides upon cycling and improves the sulfur utilization. Consequently, the binder‐free, robust, highly electrically conductive composite film exhibits outstanding electrochemical performance, including high capacities (1244–1350 mAh g^‐1^), excellent rate handling, and impressive cycling stability (0.035–0.048% capacity loss per cycle), surpassing the best MXene‐S batteries known. The fabrication of a pouch cell based on the freestanding S@Ti_3_C_2_T*_x_* film is also reported. The prototype device showcases high capacities and excellent mechanical flexibility. Considering the broad family of MXenes and their unique roles in immobilizing the polysulfides, various S@MXene composites can be similarly fabricated with promising Li^+^ storage capability and long lifetime performance.

The ever‐increasing demands for portable electronics and the emergence of electric vehicles have greatly stimulated research on energy‐storage devices.^1^, ^2^, ^3^, ^4^, ^5^, ^6^, ^7^, ^8^, ^9^, ^10^ Compared to Li‐ion batteries,^11^, ^12^, ^13^, ^14^, ^15^ rechargeable lithium‐sulfur (Li‐S) batteries exhibit clear advantages such as a theoretical energy density of 2570 Wh kg^−1^ (three to five times higher than the state‐of‐the‐art Li‐ion batteries) as well as the cost effectiveness and environmental benignity of sulfur.^16^, ^17^, ^18^, ^19^, ^20^, ^21^ However, due to the insulating nature of S, as well as the notorious shuttling effect of intermediate lithium polysulfides (Li_2_S*_x_*, *x* > 3), Li‐S batteries are still yet to be commercialized.^22^, ^23^, ^24^ Toward these challenges, much effort has been focused on developing conductive host materials (typically represented by various carbon materials)^25^, ^26^ or optimizing the electrode/electrolyte interface to facilitate the effective physical confinement or chemisorption of the Li_2_S*_x_*.^27^, ^28^, ^29^ Nevertheless, Li‐S cells still suffer from considerable decay. This is due to the intrinsic weak confinement of polar Li_2_S*_x_* in the nonpolar carbon and the depressed electron/ion transport kinetics in the polar hosts.^17^ Said otherwise, in order to develop high‐performance Li‐S batteries, ideal S hosts should be highly electrically conductive with abundant sites to immobilize the Li_2_S*_x_*.

Recently, an emerging class of 2D transition metal carbides and nitrides, so‐called MXenes, has been reported.^30^, ^31^ By selectively etching the A atomic layer from the parent MAX phase (where M represents an early transition metal, A is typically aluminum (Al) or gallium, and X is C and/or N) or other ternary layered ceramics, MXenes terminated with abundant functional groups, such as oxygen (—O), hydroxyl (—OH), and/or fluoride (—F), are obtained, which can be expressed via the formula of M*_n_*_+1_X*_n_*T*_x_* (where *n* = 1, 2, or 3 and T is a terminating group).^32^, ^33^, ^34^ In the MXene family, titanium carbide (Ti_3_C_2_T*_x_*) is the most intensively studied one and has exhibited good performances in supercapacitors,^35^, ^36^, ^37^, ^38^ Li (Na)‐ion batteries,^39^, ^40^ antibacterial coatings,^41^ electromagnetic‐interference shielding,^42^ etc. The metallic conductivity^35^, ^37^ coupled with rich surface chemistry renders Ti_3_C_2_T*_x_* an excellent host for trapping the Li_2_S*_x_*, as supported both by the density functional theory (DFT) studies and experiments.^43^, ^44^, ^45^ Nazar and co‐workers have conducted pioneering research on employing Ti_3_C_2_T*_x_*, Ti_2_CT*_x_*, and Ti_3_CNT*_x_* as cathode hosts for Li‐S batteries.^44^, ^46^ They reported that the —OH terminal groups play an important role in anchoring the Li_2_S*_x_* through the strong Ti—S bond.^44^ However, according to the X‐ray photoelectron spectroscopy (XPS), a huge peak at 459.3 eV, which is ascribed to TiO_2_, can be found in all types of MXenes,^44^, ^46^ rendering it difficult to tell if the anchoring effect of polysulfides is due to the chemisorptive nature from the MXene mediator or from the as‐formed TiO_2_ sites on the surface.

In addition, placing a physical protective barrier between the cathode and the separator has proved to be effective in suppressing the migration of Li_2_S*_x_*. This is typically realized by coating the separator with a thin conductive layer such as carbon nanotube or graphene films.^47^, ^48^ However, these carbon layers are nonpolar to the Li_2_S*_x_*. Very recently, Jin and co‐workers coated few‐layered, polar Ti_3_C_2_T*_x_* nanosheets onto the glass fiber membrane as the Li_2_S*_x_* reservoir; the Li‐S cell showed a specific capacity of 721 mAh g^−1^ after 100 cycles.^45^ Nevertheless, adding these barriers to the system not only complicates the procedures but also increases the weight of the inactive component, which inevitably compromises the cell performance. On the other hand, if the surface barrier is formed on the polar mediator in situ from the polysulfides, both the cell lifetime and S utilization will be substantially improved.

Herein, we report on the in situ formation of a thick sulfate complex layer as the protective barrier for retarding the Li_2_S*_x_* migration from the S@Ti_3_C_2_T*_x_* electrodes, thus achieving high‐capacity, ultralow‐capacity‐decay‐rate Li‐S batteries. We decorate the 2D Ti_3_C_2_T*_x_* nanosheets with nanoscale S in situ to form a viscous aqueous ink, based on which the freestanding, flexible S@Ti_3_C_2_T*_x_* electrodes were obtained without the addition of any conductive agents or polymeric binder. The polar Ti_3_C_2_T*_x_* conductive mediator endows the S@Ti_3_C_2_T*_x_* electrodes with high electrical conductivity, mechanical robustness, and a thick sulfate complex layer on the electrode surface, enabling fast electron transfer kinetics across the liquid–solid interface and suppressing the migration of Li_2_S*_x_*. In addition, we demonstrate a flexible Li‐S pouch cell based on the S@Ti_3_C_2_T*_x_* film and Li ribbon, which showcases excellent capacities under bending, indicating promise for application in future wearable electronics.

**Figure** **1**a schematically shows the preparation of the S@Ti_3_C_2_T*_x_* composite ink. The Ti_3_C_2_T*_x_* colloidal suspension, which was obtained by etching the Ti_3_AlC_2_ MAX (Figure S1, Supporting Information) in lithium fluoride‐hydrochloric acid mixture followed by bath sonication,^49^ was enriched (concentration ≈0.6 mg mL^−1^) with predominantly single‐layered flakes (d‐Ti_3_C_2_T*_x_*; Figure S2a, Supporting Information). These flakes possess an averaged thickness of 1.5 nm and a mean lateral dimension of 2.6 µm (Figure S2b,c, Supporting Information). Moreover, the absence of Al in the energy‐dispersive spectrum (EDX; Figure S2d, Supporting Information) coupled with the downshifted characteristic (0002) peak in the X‐ray diffraction patterns (Figure S3, Supporting Information) indicate the d‐Ti_3_C_2_T*_x_* flakes are clean and possess a broadened interlayer distance, in good agreement with previous reports.^49^, ^50^ Starting with the d‐Ti_3_C_2_T*_x_* colloidal suspension, sodium polysulfides (Na_2_S*_x_*) and HCOOH solution were added in sequence under stirring to form a homogeneous mixture (step I). The disproportionation reaction between Na_2_S*_x_* and HCOOH results in the in situ formation of S nanoparticles (NPs) in solution. Upon washing and centrifugation, a homogeneous S@Ti_3_C_2_T*_x_* viscous aqueous ink was obtained (step II, Figure 1b). The rheological property (Figure 1c) indicates the non‐Newtonian characteristics and shear‐thinning (pseudoplastic) behavior in this S@Ti_3_C_2_T*_x_* ink.^51^ According to the Ostwald‐de Waele power law, which can be expressed as η ∝ *kγ* ^*n*−1^ (η is the viscosity (Pa s), γ is the shear rate (s^−1^), *k* and *n* are empirical parameters, a quite small *n* (0.21) was thus obtained, indicative of a great degree of shear‐thinning behavior. The high concentration and viscosity (≈20 mg mL^−1^ and 12.4 Pa s, respectively) enabled the ink to be arbitrarily painted on various substrates such as Celgard membranes (Figure 1d), paper, and stainless steel (Figure S4a,b, Supporting Information). Importantly, this viscous aqueous ink can be directly slurry‐casted onto Al foil using an industrially compatible doctor‐blade technique (Figure 1e) without the need of polymeric binder or carbon black or organic solvent. This is of significant importance and it allows the whole procedure to be simple and environmentally friendly. In this study, we vacuum‐filtrated the viscous ink and obtained the freestanding films for the characterization (Figure S4c, Supporting Information).

**Figure 1:**
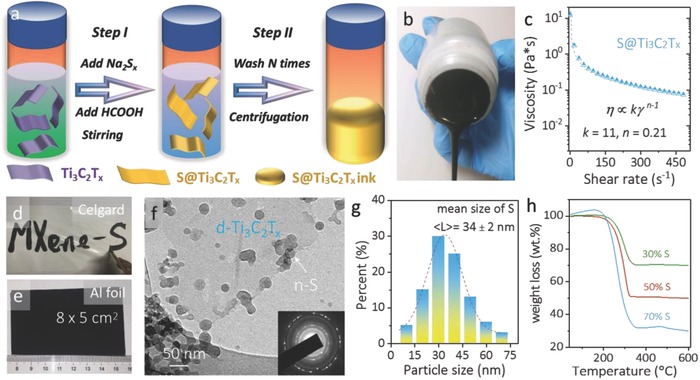
a) Schematic preparation and b) optical image of S@Ti_3_C_2_T*_x_* ink, showing its viscous nature. c) Viscosity of S@Ti_3_C_2_T*_x_* ink plotted as a function of shear rate. d) Handwriting on Celgard membrane and e) doctor‐blade casting on Al foil using the S@Ti_3_C_2_T*_x_* ink. f) TEM image of S@Ti_3_C_2_T*_x_* ink. The inset is the selected area electron diffraction (SAED) pattern. g) Histogram of sulfur NPs in the S@Ti_3_C_2_T*_x_* ink. h) Thermogravimetric profiles of S@Ti_3_C_2_T*_x_* films with different sulfur loadings.

Due to the hydrophilic nature of d‐Ti_3_C_2_T*_x_*, the nanosheets can effectively disperse S NPs during synthesis.^52^ In addition, the electronegative surface groups (such as —O and —OH) on the d‐Ti_3_C_2_T*_x_* flakes provide abundant anchoring sites for the S NPs through the electrostatic interaction, as supported by the zeta‐potential results (Figure S5, Supporting Information). Such an interaction limits the self‐growth of S^52^, ^53^ and results in nanoscale S decorated onto/between the nanosheets. This is confirmed by the transmission electron microscopy (TEM) images (Figure 1f and Figure S6, Supporting Information). On the other hand, when the guiding/limiting effect of d‐Ti_3_C_2_T*_x_* was absent, S agglomerates were found with a mean size of ≈2.6 ± 0.1 µm (Figure S7, Supporting Information), in sharp contrast with the in situ formed crystalline S (≈34 ± 2 nm; Figure 1g). By simply adjusting the mass ratio of Na_2_S*_x_* and HCOOH to d‐Ti_3_C_2_T*_x_*, S@Ti_3_C_2_T*_x_* inks and composite films with desired S loading were obtained (Figure 1h). The slight mass increase in the 70% S sample might come from the Ti_3_C_2_T*_x_* oxidation triggered by the O_2_ impurity in the new Ar tank, as the oxidation is a mass increasing reaction. Composites with medium (50%) and high (70%) S loading were chosen for further studies. The sulfur mass loading in the composite reached a decent value, 2.49 ± 0.11 mg cm^−2^.

The scanning electron microscopy (SEM) images in **Figure** **2**a,b suggest a compact morphology in the 70% S@Ti_3_C_2_T*_x_* electrode with Ti_3_C_2_T*_x_* flakes continuously crosslinked to each other (Figure 2c). In addition, S NPs are uniformly distributed, as shown in the elemental mapping (insets of Figure 2a,b) and cross‐sectional TEM image (Figure 2d). Consequently, the composite showcases a quite low specific surface area (1.2 m^2^g^−1^; Figure S8a, Supporting Information). Such an architecture not only provides an advanced electrically conductive network for the high‐rate charge–discharge, but also endows the electrode with mechanical robustness. Figure 2e indicates that the freestanding 70% S@Ti_3_C_2_T*_x_* film (20 µm in thickness) can be convexly or concavely bended without any cracking, indicative of good mechanical flexibility. The 70% S@Ti_3_C_2_T*_x_* film displays very high tensile strength (≈12.9 MPa) and Young's modulus (≈19.2 GPa; Figure 2f). In addition, the tensile strain of the film reaches 0.8%, higher than that of graphene oxide paper (<0.6%)^52^, ^54^ but lower than that of Ti_3_C_2_T*_x_* paper (≈6%).^55^ This is because the large amount of nanocrystalline S in the composite (Figure S8b, Supporting Information) compromises the film's stretchability. The electrical conductivity also reduces from 1984 S cm^−1^ in the Ti_3_C_2_T*_x_* film to 745 and 175 S cm^−1^ in the 50% S and 70% S@Ti_3_C_2_T*_x_* films, respectively (Figure 2g). Despite that, the conductivity in the 70% S@Ti_3_C_2_T*_x_* film is more than one order of magnitude higher than that of graphene (8 S cm^−1^)^48^ or 1T‐MoS_2_ (10 S cm^−1^)^56^ based electrodes. The excellent electrical conductivity and good mechanical flexibility in the freestanding S@Ti_3_C_2_T*_x_* films are critical to achieve a high‐performance, robust Li‐S battery.

**Figure 2:**
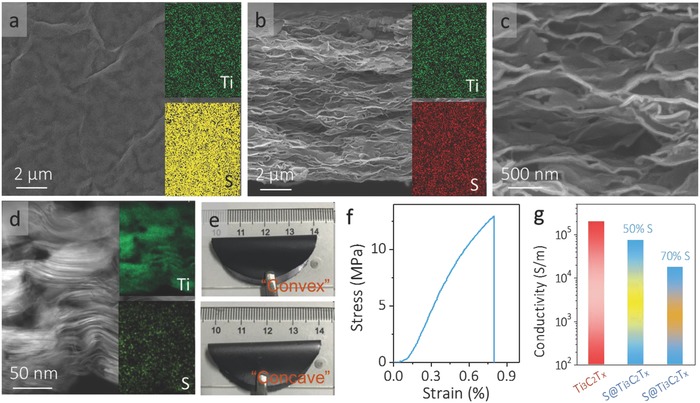
SEM images of a) top view and b) cross‐section of S@Ti_3_C_2_T*_x_* film. The insets are EDX mapping images. c) Higher magnification of the cross‐section SEM image. d) Cross‐sectional TEM image of S@Ti_3_C_2_T*_x_* electrode. The insets are elemental mapping of Ti and S. e) Photographs of freestanding S@Ti_3_C_2_T*_x_* film when bent convexly (up) and concavely (down), showing good mechanical flexibility. f) Stress–strain curve of the 70% S@Ti_3_C_2_T*_x_* film. g) Electrical conductivity of Ti_3_C_2_T*_x_* film and S@Ti_3_C_2_T*_x_* films with different S loadings.

We assembled Li‐S coin cells using the freestanding S@Ti_3_C_2_T*_x_* films as the cathode and Li foil as the anode. In the cyclic voltammograms (CVs) of 70% S@Ti_3_C_2_T*_x_* (**Figure** **3**a), the cathodic peaks at 2.2–2.4 V and 1.9–2.1 V can be attributed to the formation of long‐chain soluble polysulfides and short‐chain insoluble Li_2_S, respectively.^57^ Due to the formation of solid–electrolyte interphase, the first cycle CV showcases more intense cathodic peaks compared to the subsequent cycles. The anodic peak at 2.2–2.5 V corresponds to the formation of elemental sulfur.^52^ After the initial two cycles of stabilization, the anodic peaks gradually shift to lower potential, indicating an improved coulombic efficiency. To highlight the synergistic effect between the in situ formed S NPs and Ti_3_C_2_T*_x_* host, a conventional composite electrode was fabricated (Figure S9, Supporting Information). Figure 3b and Figure S10 (Supporting Information) compare the stabilized galvanostatic charge–discharge (GCD) curves and the first‐cycle coulombic efficiency in these electrodes. The smaller GCD polarization (30 mV), higher capacity (1250 mAh g^−1^), and coulombic efficiency (90.6%; Figure S10, Supporting Information) in the 70% S@Ti_3_C_2_T*_x_* indicate that the electrochemical kinetics on the polar, conductive Ti_3_C_2_T*_x_* host are quite favorable. On the other hand, apparent phase separation and S agglomerates were observed in the physically mixed sample, which demonstrated a rough electrode surface (Figure S9b,c, Supporting Information). Consequently, the electron transport as well as ion diffusion kinetics is suppressed, resulting in lower capacity (990 mAh g^−1^) and initial coulombic efficiency (80.7%; Figure S10, Supporting Information).

**Figure 3:**
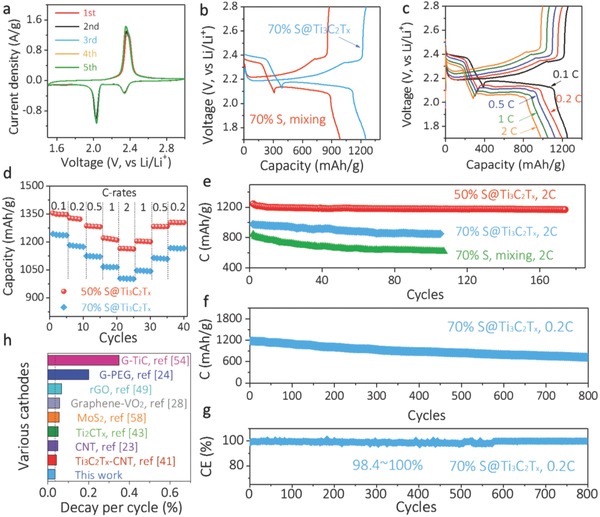
Electrochemical characterization of S@Ti_3_C_2_T*_x_* electrodes. a) CV curves of 70% S@Ti_3_C_2_T*_x_* at 0.1 mV s^−1^ at different cycles. b) GCD profiles of 70% S@Ti_3_C_2_T*_x_* in comparison to the Ti_3_C_2_T*_x_*–S mixture. c) GCD curves of 70% S@Ti_3_C_2_T*_x_* at various C‐rates. d) Rate handling e) cycling performances of S@Ti_3_C_2_T*_x_* cathodes (at 2 C) in comparison to the Ti_3_C_2_T*_x_*–S mixture. f) Long‐term cycling and g) coulombic efficiency of 70% S@Ti_3_C_2_T*_x_* at 0.2 C. h) Comparison of capacity decay rate (per cycle) of this work to reported Li‐S cathodes. The dashed line corresponds to the decay rate of 0.035%.

The GCD curves of 50% S and 70% S@Ti_3_C_2_T*_x_* at various C‐rates are shown in Figure S11 (Supporting Information) and Figure 3c, respectively. Both electrodes exhibit two discharge plateaus, corresponding to the conversion of elemental sulfur to soluble polysulfides (2.3–2.4 V) and insoluble Li_2_S (2.1–2.2 V). At 0.1 C (1 C = 1675 mA g^−1^), the 70% S@Ti_3_C_2_T*_x_* exhibits a capacity of 1244 mAh g^−1^ and maintains 1004 mAh g^−1^ (capacity retention of 80.7%) as the C‐rate increased by 20‐fold, demonstrating excellent rate capability (Figure 3d). When the C‐rate switches back to 0.2 C, a tiny capacity difference is observed (≈0.7%), further evidence of reversible electrochemical reactions that occurred at the liquid–solid interface. The 50% S@Ti_3_C_2_T*_x_* electrode displays even higher capacities (1350 mAh g^−1^ at 0.1 C) and better rate capability (86% capacity retention as increasing the C‐rate by 20‐fold) compared to the 70% S@Ti_3_C_2_T*_x_* electrode. It is noteworthy that our S@Ti_3_C_2_T*_x_* electrodes have greatly exceeded the capacities of other reported systems at various C‐rates, such as graphene nanoscrolls,^58^ graphene paper,^59^ carbon nanotubes (CNT),^60^ or their composites^48^ (Figure S12, Supporting Information). Although reduced graphene oxide‐S freestanding paper exhibited quite similar capacities and rate handling to our 70% S@Ti_3_C_2_T*_x_*, we note that their performance was achieved at a lower S loading (60%).^52^

The lifetime performance of S@Ti_3_C_2_T*_x_* films is shown in Figure 3e and Figure S13 (Supporting Information). The initial capacity of 50% S@Ti_3_C_2_T*_x_* is 1246 mAh g^−1^, and maintains 1170 mAh g^−1^ after 175 cycles at 2 C, suggesting an ultralow capacity decay rate (0.035% per cycle). At the same rate (2 C), the 70% S@Ti_3_C_2_T*_x_* delivers 850 mAh g^−1^ after 100 cycles (Figure 3e and Figure S13b, Supporting Information). At a slower charging–discharging rate (0.2 C), the 70% S@Ti_3_C_2_T*_x_* electrode exhibits an initial capacity of 1184 mAh g^−1^ and maintains 724 mAh g^−1^ after cycling for 800 times (Figure 3f), corresponding to a low capacity decay rate (0.048%). The coulombic efficiency varies from 98.4% to 100% (Figure 3g), indicating quite reversible electrochemical reactions have been achieved in this high S loading electrode during cycling. In contrast, the physically mixed Ti_3_C_2_T*_x_*–S composite electrode demonstrates a lower initial capacity of 917 mAh g^−1^ and decays to 617 mAh g^−1^ after 107 cycles (Figure 3e). This can be attributed to the inferior electron transport kinetics to the S@Ti_3_C_2_T*_x_* and the larger charge‐transfer resistance across the liquid–solid interface (Figure S14, Supporting Information).

We further compared the capacity decay rate of S@Ti_3_C_2_T*_x_* to other reported systems (Figure 3h). Among various sulfur hosts, such as TiC@graphene,^57^ graphene‐poly(ethylene glycol),^25^ graphene/VO_2_,^29^ CNTs,^24^ MoS_2_,^61^ and MXenes (Ti_2_CT*_x_* and Ti_3_C_2_T*_x_*‐CNT),^44^, ^46^ our S@Ti_3_C_2_T*_x_* electrode demonstrates the lowest capacity decay rate. We believe several factors could be responsible for the excellent electrochemical performance: (1) The crosslinked network ensures rapid electron transport and ion diffusion kinetics; (2) The S NPs intimately decorate the conductive Ti_3_C_2_T*_x_* mediator, allowing improved S utilization and reversible redox reactions; (3) The polar host facilitates the direct nucleation of Li_2_S and suppresses the polysulfides shuttle.

To examine the interaction between Li_2_S*_x_* and Ti_3_C_2_T*_x_*, we immersed the Ti_3_C_2_T*_x_* freestanding film into the representative polysulfide solution (Li_2_S_4_) inside an Ar‐filled glove box. The bright yellow solution quickly faded and became almost colorless after 1 h interaction (Figure S15, Supporting Information), a direct proof of the strong chemisorptive nature of the polar, conductive mediator. We further performed first‐principle DFT calculations to illustrate the nature and bonding strength between Ti_3_C_2_T*_x_* and Li_2_S_4_. For simplicity, we assume that the Ti_3_C_2_T*_x_* nanosheets are terminated with either —OH or —O groups. The significant distortion of the Li_2_S_4_ molecule confirms the strong capability of Ti_3_C_2_T*_x_* in immobilizing the polysulfides (**Figure** **4**a–c). The hydroxyl‐terminated Ti_3_C_2_T*_x_* showcases the highest binding energy (≈14.0 eV; Figure 4d), which is in the same range that Nazar and co‐workers reported,^44^ suggesting the —OH groups play an important role in trapping the Li_2_S_4_.

**Figure 4:**
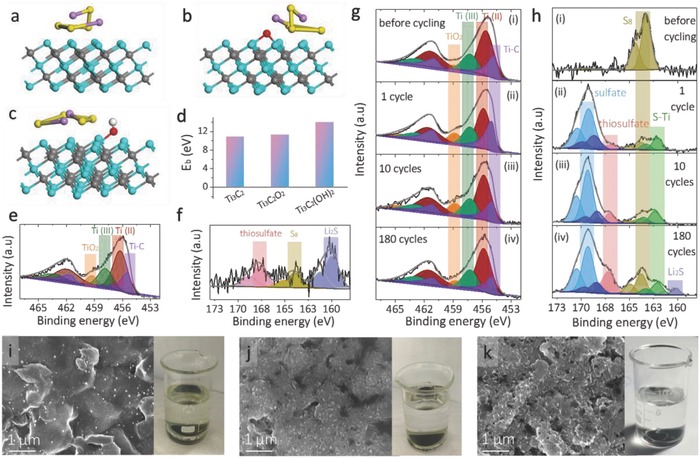
Adsorption configuration of Li_2_S_4_ on a) Ti_3_C_2_, b) Ti_3_C_2_O_2_, and c) Ti_3_C_2_(OH)_2_. d) Binding energy between Li_2_S_4_ and Ti_3_C_2_ with different terminal groups. XPS analysis of e) Ti 2p and f) S 2p spectra in Ti_3_C_2_T*_x_* after interacting with Li_2_S_4_. g) Ti 2p and h) S 2p in the fresh S@Ti_3_C_2_T*_x_* film and cycled electrodes for different times. Top‐view SEM images of the S@Ti_3_C_2_T*_x_* electrode after i) 1 cycle, j) 10 cycles, and k) 50 cycles. The right panels are the corresponding photographs of cycled electrodes immersed in the solvent, showing the different extent of polysulfide diffusion.

The surface environment on the Ti_3_C_2_T*_x_* mediator was further examined using XPS. After contact with the polysulfide solution, the Ti 2p spectrum is roughly similar to that of the fresh one (Figure 4e and Figure S16, Supporting Information). We note that it is hard to conclude whether the Ti—S bond formed or not after the interaction based on the Ti 2p spectrum alone, as the Ti‐S (455.6 eV) overlaps with the Ti‐C (455.1 eV) peak. In the S 2p spectrum, peaks corresponding to thiosulfate (167.6 eV),^44^ Li_2_S (159.8 eV), as well as elemental sulfur (S_8_) are observed; no S—Ti bond is found (Figure 4f). The O1s spectrum in Figure S17 (Supporting Information) indicates that the —OH groups were reduced by the Li_2_S_4_ and formed thiosulfate as a result. We propose the in situ formed thiosulfate species can function as a protective layer that facilitates the direct nucleation of Li_2_S on the mediator, suppresses the Li_2_S*_x_* shuttle kinetics, and improves the S utilization, leading to enhanced rate capability and long lifetime in the S@Ti_3_C_2_T*_x_* electrodes.

To check the above hypothesis, we analyzed the XPS spectra of the electrode after different cycles, as shown in Figure 4g,h. The absence of a S—Ti bond (Figure 4h (i)) in the fresh 70% S@Ti_3_C_2_T*_x_* electrode indicates that no chemical bond was formed during the in situ solution growth of sulfur at room temperature. The Ti 2p spectra under different cycles are similar (Figure 4g). After one cycle, dominant sulfate/thiosulfate complex (168.5 and 169.4 eV) and S—Ti bond (162.3 eV) are found (Figure 4h (ii)), the latter was formed due to the bonding of either thiosulfate or Li_2_S*_x_* to the exposed Ti atoms after the cleavage of the hydroxyl groups by the polysulfides, according to Nazar and co‐workers.^44^ The S 2p spectrum is similar after ten more cycles (Figure 4h (iii)). After 180 cycles, the total intensity of sulfate/thiosulfate complex increased considerably coupled with some elemental S (S_8_), implying that the sulfate layer kept growing during cycling. The good coverage of such a protective layer allows efficient immobilization of Li_2_S*_x_*. The tiny Li_2_S peak in Figure 4h (iv) is probably a result of the locally diffused Li_2_S*_x_* upon long‐term cycling. Predominant peaks from the sulfate complex as well as S—Ti bond are also observed in the 50% S@Ti_3_C_2_T*_x_* after one cycle (Figure S18, Supporting Information), indicating that the sulfate layer tightly covered the nanosheet backbone and is independent of the sulfur loadings.

The SEM images of the 70% S@Ti_3_C_2_T*_x_* electrodes after different cycles were examined. After one cycle, the smooth surface of the fresh electrode becomes rougher and is decorated with a layer of nanoparticles (Figure 4i, left), which are most probably the sulfates. These particles grow larger upon another ten cycles, forming a continuous layer covered on the electrode surface (Figure 4j, left). After 50 cycles, an even thicker sulfate layer made of larger particles is observed (Figure 4k, left), in good agreement with the XPS results. If the formation of the sulfate layer is the main reason for suppressing the polysulfides shuttle effect, then we would expect a more complete confinement in the electrodes with a higher cycle number, as the sulfate layer is thicker. Therefore, we disassembled the cells after discharging to 2.1 V and immersed the electrodes, which were cycled for different times, into the solvent (inside the Ar‐filled glove box). No leakage of yellow media is found in the electrode with a higher cycle number (50 cycles; Figure 4k, right), in sharp contrast to the ones with a lower cycle number. For example, light and bright‐yellow liquid were observed in electrode after cycling for one and ten times, respectively (Figure 4i,j, right). These results suggest that a thick sulfate complex layer after 50 cycles is much more beneficial than a thinner layer in trapping the soluble polysulfides. In other words, compared to the S—Ti bond, the thick layer of sulfate complex is more responsible for the efficient immobilization of Li_2_S*_x_* and the excellent lifetime of the S@Ti_3_C_2_T*_x_* electrode.

Although the intrinsic formation of the sulfates is quite complex and beyond the scope of the DFT calculations, we postulate three possible steps to describe the process (**Scheme** **1**). First, the as‐formed Li_2_S*_x_* are chemisorbed onto the polar Ti_3_C_2_T*_x_* mediator and strongly bond to the —O and —OH groups. Second, the terminal groups are cleaved by reacting with Li_2_S*_x_*, forming thiosulfate and exposing Ti atoms. Third, the thiosulfate further reacts with Li_2_S*_x_* and forms a sulfate protective barrier, under which the exposed Ti atoms bond to Li_2_S*_x_* through the Lewis acid–base interactions.^44^ Consequently, the combination of in situ formed sulfate complex layer and the bare Ti sites efficiently entraps the polysulfides during cycling.

**Scheme 1:**
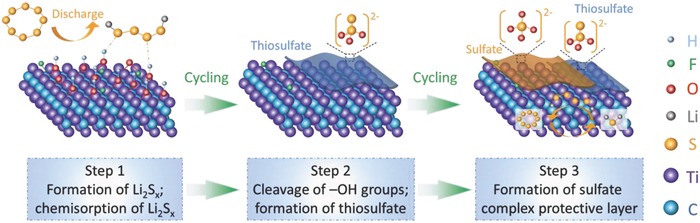
Schematic demonstration of Ti_3_C_2_T*_x_* entrapping the polysulfides by forming a sulfate complex protective barrier.

Finally, to show the potential of our S@Ti_3_C_2_T*_x_* composite for future wearable, flexible Li‐S batteries, as a proof of concept, we assembled a pouch cell by sandwiching 70% S@Ti_3_C_2_T*_x_*, separator and lithium ribbon in sequence, and sealed within a commercial plastic bag. Note that no extra force was applied on the cell during either assembly or testing. **Figure** **5**a,b shows photographs of the Li‐S pouch cell under flat and bending states, respectively. The bent cell showcases an initial capacity of 1263 mAh g^−1^ at 0.5 C (Figure 5c), higher than that of the flat cell (1124 mAh g^−1^), which can be attributed to its looser cell configuration and less efficient charge transport. After five cycles, the pouch cell still showcases a high capacity (1119 mAh g^−1^) in the bent state, while the flat cell decays faster (903 mAh g^−1^; Figure 5c). We believe that, through improving the cell packaging/sealing, a much better cycle life in this S@MXene cell is expected. To demonstrate the real application of our S@Ti_3_C_2_T*_x_* pouch cells, both the flat and bent cells were used to power an “M‐S”‐shaped string made of 37 light‐emitting diodes (LEDs). As demonstrated in Figure 5d,e and Video S1 (Supporting Information), the LEDs are brightly lit by the Li‐S cell under flat or repeated bent states at various degrees, indicating the great potential of our pouch cells for powering future flexible, wearable electronics. By further optimization and engineering, such as cathode mass loading, and/or the use of separator/electrolyte additives, we believe the performance of S@Ti_3_C_2_T*_x_* composite can be pushed further.

**Figure 5:**
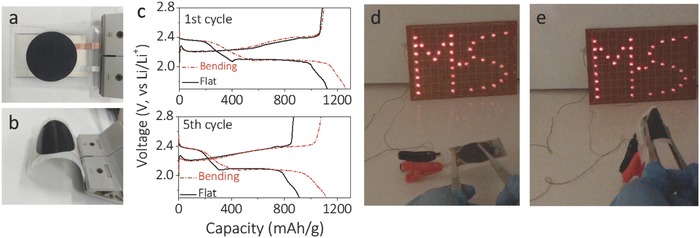
Photographs of Li‐S pouch cells based on the 70% S@Ti_3_C_2_T*_x_* and Li ribbon under a) flat and b) bent states. c) GCD profiles of the Li‐S pouch cells tested under flat and bent states. d,e) Application of the pouch cells. The optical images show an “M‐S”‐shaped string containing 37 LEDs powered by the pouch cell under d) flat and e) bent states.

In summary, we have shown the fabrication of S@Ti_3_C_2_T*_x_* viscous aqueous ink and binder‐free, robust, highly electrically conductive films through a facile slurry‐casting or filtration. The S@Ti_3_C_2_T*_x_* freestanding films have exhibited high capacities (1350 mAh g^−1^ and 1244 mAh g^−1^ in 50% S and 70% S, respectively), excellent rate handling, and ultralow capacity decay rate (0.035% per cycle in 50% S after 175 cycles and 0.048% per cycle in 70% S after 800 cycles). The impressive electrochemical performance can be well attributed to the synergistic effects between sulfur NPs and conductive, polar Ti_3_C_2_T*_x_* backbone, where the electron transport and ion diffusion kinetics have been substantially enhanced. Importantly, we have found that the polar Ti_3_C_2_T*_x_* efficiently chemisorbs the soluble polysulfides and converts them into thiosulfate and a subsequent sulfate complex. The in situ formed sulfate complex layer acts as a protective barrier for blocking the polysulfides migration, leading to the enhancement of S utilization, capacities, rate handling, and long‐term cycling stability in the S@Ti_3_C_2_T*_x_* cathode. The robust nature together with the high‐capacity, high‐rate response of S@Ti_3_C_2_T*_x_* renders the Li‐S pouch cells with promising preliminary results, which will enable future applications in wearable and flexible electronics.

## Experimental Section

Experimental details including MXene synthesis, ink formation, films fabrication, and their physical/electrochemical characterizations are listed in the Supporting Information.

## Conflict of Interest

The authors declare no conflict of interest.

## Supporting information

Supplementary

Supplementary
