# Supplementary material for: In Situ Formed Protective Barrier Enabled by Sulfur@Titanium Carbide (MXene) Ink for Achieving High‐Capacity, Long Lifetime Li‐S Batteries
Source: Adv Sci (Weinh). 2018 Jul 4;5(9):1800502. doi: 10.1002/advs.201800502 (PMC6145260; doi:10.1002/advs.201800502)
Supplement: Supplementary file 1 — Supplementary [file ADVS-5-1800502-s002.pdf]

Copyright WILEY-VCH Verlag GmbH & Co. KGaA, 69469 Weinheim, Germany, 2018.

ADVANCED SCIENCE Open Access

Supporting Information

for *Adv. Sci.,* DOI: 10.1002/advs.201800502

In Situ Formed Protective Barrier Enabled by Sulfur@Titanium Carbide (MXene) Ink for Achieving High-Capacity, Long Lifetime Li-S Batteries

Huan Tang, Wenlong Li, Limei Pan, Conor P. Cullen, Yu Liu, Amir Pakdel, Donghui Long, Jian Yang, Niall McEvoy, Georg S. Duesberg, Valeria Nicolosi,* and Chuanfang (John) Zhang**

Copyright WILEY-VCH Verlag GmbH & Co. KGaA, 69469 Weinheim, Germany, 2016.

Supporting Information

In-situ **Formed Protective Barrier Enabled by Sulfur@Titanium Carbide (MXene) Ink for Achieving High-Capacity, Long-Lifetime Li-S Batteries**

By Huan Tang, Wenlong Li, Limei Pan, Conor P. Cullen, Yu Liu, Amir Pakdel, Donghui Long, Jian Yang, Niall McEvoy, Georg S. Duesberg, Valeria Nicolosi* and Chuanfang (John) Zhang**

Synthesis of multi-layered and delaminated $Ti_3C_2T_x$ MXenes

Enhanced exfoliation and delamination of two-dimensional $Ti_3C_2T_x$ MXene using HCl+LiF etchant were reported in detail in our previous work.[1] Typically, 1 g of Ti_3AlC_2 powder (particle size < 38 μm) was gradually added into a mixed solution composed of LiF (0.67 g) and HCl (10 mL, 6 M). The reaction was held at 35 °C for 24 h under continuous stirring. The resultant product was washed with ethyl alcohol *via* centrifugation until the pH of the supernatant reached ~6. The sediment was vacuum filtrated through a nylon membrane and naturally dried and then denoted as multi-layered MXene (m-$Ti_3C_2T_x$). To delaminate the nanosheets, the m-$Ti_3C_2T_x$ powder was dispersed in deionized water (DI-water) with an initial concentration of 3.5 mg/mL. After sonication for 1 h followed by centrifugation at 3500 rpm for 1 h, the stable colloidal suspension, enriched with delaminated, few-layered $Ti_3C_2T_x$ nanosheets, was obtained with a concentration of ~0.6 mg/mL. Upon freeze-drying of the suspension, the powder of the delaminated $Ti_3C_2T_x$ nanosheets was obtained, which was denoted as d-$Ti_3C_2T_x$.

Synthesis of Na_2S_4 solution

In a typical preparation of sodium polysulfide (Na_2S_4) solution, firstly, 24 g of $Na_2S·9H_2O$ was dissolved in 1000 mL DI-water under magnetic stirring at room temperature for 0.5 h. Then 9.6 g sulfur was added to the above solution under stirring until a clear solution was formed. The resultant Na_2S_4 was used to react with HCOOH to produce S NPs, which decorated the $Ti_3C_2T_x$ nanosheets. For comparison, pure sulfur particles were also prepared when no $Ti_3C_2T_x$ was added.

Aqueous ink preparation

The S@$Ti_3C_2T_x$ aqueous ink was prepared as follows. Typically, 100 mL of $Ti_3C_2T_x$ aqueous solution (0.6 mg/mL) was mixed with 73 mL of diluted Na_2S_4 solution (0.02 M) and then stirred for 20 min. Thereafter, 0.2 M formic acid solution was added dropwise into the above suspension until pH <7. During this process, S nanoparticles were formed *in situ* through the disproportionation reaction between Na_2S_x and HCOOH, homogenously nucleated and decorated the $Ti_3C_2T_x$ nanosheets. The above suspension was stirred for another 20 min and then allowed to stand still for 30 min, followed by centrifugation at 5000 rpm for 10 min. After decanting 80% of the supernatant and redispersing the sediment, a black viscous ink was obtained, which can be painted on various substrates including Celgard membrane, paper, stainless steel and Al foil. To prepare electrodes for Li-S batteries, the ink was directly vacuum-filtrated through the polyethylene membrane (Celgard 3501, USA) and then naturally dried. The freestanding film was denoted as 70% S@$Ti_3C_2T_x$. By simply adjusting the mass ratio of Na_2S_x and HCOOH to d-$Ti_3C_2T_x$ solution, the S loading can be regulated from 30 to 70%.

Materials characterization

The morphologies of the samples were characterized by scanning electron microscopy (FE-SEM, Hitachi SU8010). Elemental mapping analysis was carried out using a scanning electron microscope (SEM, FEI Q300). X-ray diffraction (XRD) measurements were conducted using a Rigaku Smartlab (Tokyo, Japan) diffractometer with Cu-Kα radiation

(λ=1.54178 Å) under a step scan of 0.02° and step time of 0.5 s. Atomic force microscopy (AFM) was performed on a MultiMode 8 microscope (Bruker, Germany). X-ray photoelectron spectroscopy (XPS, ULVAC-PHI, Japan) was performed using a PHI 5000 Versa Probe spectrometer equipped with a monochromatic Al-Ka (1486.6 eV) X-ray source. Nitrogen adsorption and desorption isotherms were obtained on an ASAP 2020 M analyzer (Micromeritics, USA). The BET specific surface areas of the samples were calculated using 5 points and the pore size distribution was derived from a density functional theory (DFT) model. The electrical conductivity of the samples was measured by a four-point-probe resistivity tester (RTS-9, Guangzhou four-probe Co. Ltd, China). The stress-strain test of the sample was performed on a universal tensile tester (ESM301, USA). The sulfur content was determined by thermal gravimetric analysis (TGA, Netzsch, STA-449C) from 25 to 500 °C under argon atmosphere. The heating rate was controlled to 10 °C/min. The rheological behavior of the $S@Ti_3C_2T_x$ slurry was determined using an intelligent viscometer (RheoWin HAAKE Viscotester iQ Air, Germany).

DFT calculations

We used quantum mechanics (QM) to calculate the binding energy between Li_2S_4 and MXenes with different terminal groups. The binding energy (E_b) is calculated by

$$E_b = E_{MXene} + E_{Li2S4} - E_{MXene+Li2S4} \quad (1)$$

where $E_{MXene+Li2S4}$, E_{MXene} and E_{Li2S4} stand for the energy of Li_2S_4-MXene composite, MXene and Li_2S_4, respectively. The QM calculation is implemented in the Dmol^3 module of Material Studio software. The exchange correlation functional was modeled by the Perdew-Burke-Ernzerhof (PBE) method. The Grimme method was employed to calculate the dispersion correction, effective core potentials were used to model the core-electron interactions, and the one body wave functions were numerically expanded by the DND3.5 basis set. In the computation, MXene and Li_2S_4 were placed in a periodic box with parameters a = b = 0.922

nm, c = 1.856 nm $\alpha = \beta$ = 90°, γ = 120°. The initial conformation of the MXene and Li_2S_4 were obtained by molecular mechanics (MM) optimization, which was implemented by the Forcite module with the Universal force field (UFF). The self-consistent iteration in QM is considered to converge when the change of energy is lower than 2.72×10^{-4} eV while the optimization is considered to converge when the change of energy is lower than 5.44×10^{-4} eV, the force lower than 0.33 nN and the displacement lower than 5×10^{-4} nm.

Assembly of Li-S batteries

All the S@$Ti_3C_2T_x$ cathodes were obtained by vacuum-filtration. Both coin-type and pouch half cells were assembled. The S@$Ti_3C_2T_x$ film was directly punched into circular pellets (diameter of 13 mm) as the cathode. For the purpose of comparison, a $Ti_3C_2T_x$-S-mixture cathode was fabricated by physically mixing the S particles with freeze-dried $Ti_3C_2T_x$ powder and poly(vinylidene fluoride) binder in a weight ratio of 7:2:1 in dimethylformamide. The slurry was then cast onto Al foil, followed by drying at 60 °C in vacuum. Lithium foils and Celgard 3501 were used as the anode and separator, respectively. The electrolyte was 1 M lithiumbis (trifluoromethanesulfonyl) imide (LiTFSI) in 1,3-dioxolane (DOL) and 1,2-dimethoxyethane (DME) (1:1, vol) with 1 wt% $LiNO_3$. CR-2032 coin cell batteries were assembled in an argon-filled glove box (Mikrouna Universal 2440/750). Before electrochemical tests, the cells were aged for 10 h to ensure a fully wetted electrode. As for the pouch half cell, as a prototype, the entire vacuum-filtrated S@$Ti_3C_2T_x$ film was used as the cathode and lithium ribbon as the anode. The electrolyte and separator for the pouch cell were kept the same as those of coin cells. To facilitate the connection to external cables, Al and copper foil were attached to the cathode and anode, respectively. All the components were compactly stacked and encapsulated in a self-sealing plastic bag under vacuum inside the Ar-filled glove box.

***Electrochemical measurements*:** To evaluate the electrochemical performance of the S@$Ti_3C_2T_x$ cathode, charge/discharge tests were conducted using a Neware cell test system

(CT-3008, Shenzhen, China) in the range of 1.7 to 2.8 V. The galvanostatic charge/discharge (GCD) tests were carried out at 0.1 C (1 C = 1675 mA/g), 0.2 C, 0.5 C, 1 C, 2 C in a potential window of 1.7-2.8 V (vs Li/Li^+). The cyclic voltammetry (CV) tests were performed from 1.5 to 3 V (vs Li/Li^+) at 0.1 mV s^{-1}. Electrochemical impedance spectroscopy (EIS) was measured at open circuit potential in the frequency range of 10 mHz to 100 kHz with an AC voltage amplitude of 10 mV. Both CV and EIS were conducted on a CHI660C electrochemical workstation (Shanghai Chenhua, China). Cycling performance of the Li-S half-cell was performed at 0.2 and 2 C for 800 cycles. In addition, some cells were disassembled after different cycles with electrode cleaned in the 1,3-dioxolane (DOL) and 1,2-dimethoxyethane (DME) (1:1, vol) mixed solvent before further characterization .

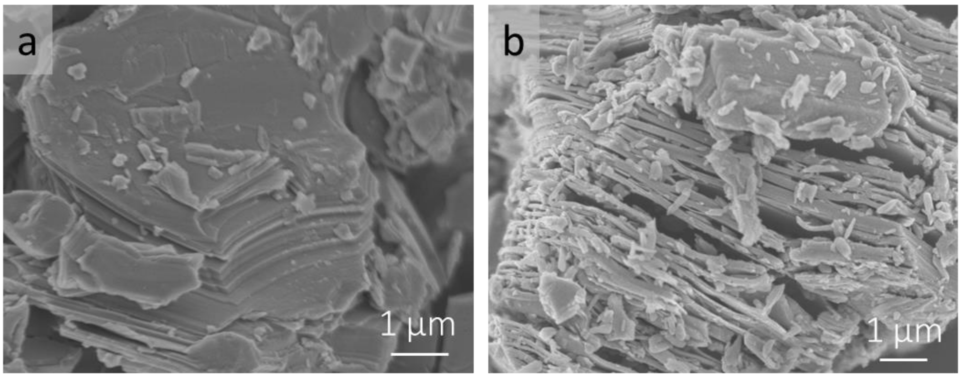

Figure S1. SEM images of (a) Ti_3AlC_2 MAX and (b) multi-layered MXene (m-$Ti_3C_2T_x$). The apparent gap among the sheets in (b) suggest successful etching of the MAX phase in the LiF-HCl solution. Moreover, the accordion-like morphology of the m-$Ti_3C_2T_x$ in (b) is similar to previous reports.[2,3]

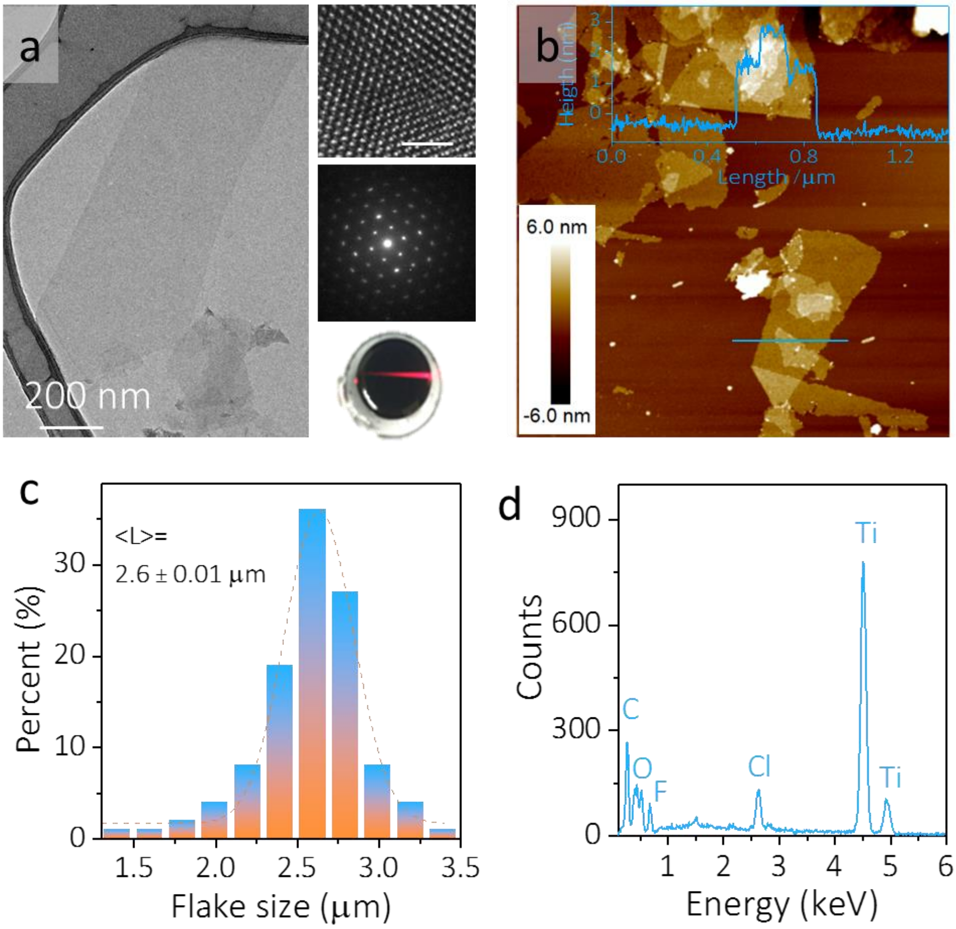

Figure S2. (a) TEM image of $Ti_3C_2T_x$ flakes. Insets: Top: high-resolution TEM (HRTEM, scale bar: 2 nm); Middle: selected-area electron diffraction (SAED); Bottom: Tyndall effect of the d-$Ti_3C_2T_x$ colloidal solution. (b) AFM image of a $Ti_3C_2T_x$ flake. Inset is the height profile. (c) Flake lateral size histogram. (d) EDX pattern of $Ti_3C_2T_x$ nanosheets.

The monolayered nanosheets possess a clean surface with an averaged thickness ~1.5 nm (a, b, d), agreeing well with previous reports.[4,5] These flakes are highly crystalline with the hexagonal atomic structure, as seen in the HRTEM and SAED in (a). In addition, these nanosheets possess a mean lateral size of ~2.6 μm (c). The high aspect ratio (~1720) endows the nanosheets a high flexibility, which is beneficial for the S@MXene electrodes.

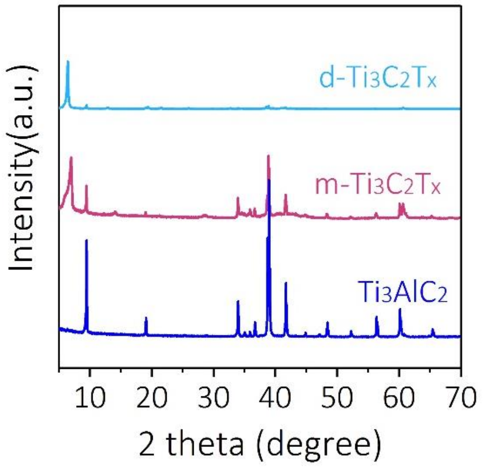

Figure S3. XRD patterns of Ti_3AlC_2, m-$Ti_3C_2T_x$ and delaminated (d-) $Ti_3C_2T_x$.

The XRD patterns of three samples indicate the successful etching and delamination of the nanosheets. Compared to the MAX phase, the existence of the (0002) peak in m-$Ti_3C_2T_x$ suggest the presence of MXene. In addition, apparent peaks corresponding to the MAX phase can be found in m-$Ti_3C_2T_x$, suggesting that the etching was incomplete. This could be improved by adding more LiF and HCl as detailed in a previous report.[4,6]

Upon sonication, delamination of m-$Ti_3C_2T_x$ occurred. This is best evidenced by the downshifting of the (0002) peak. Only the (00*l*) peaks are found, suggesting the layered structure of the MXene.

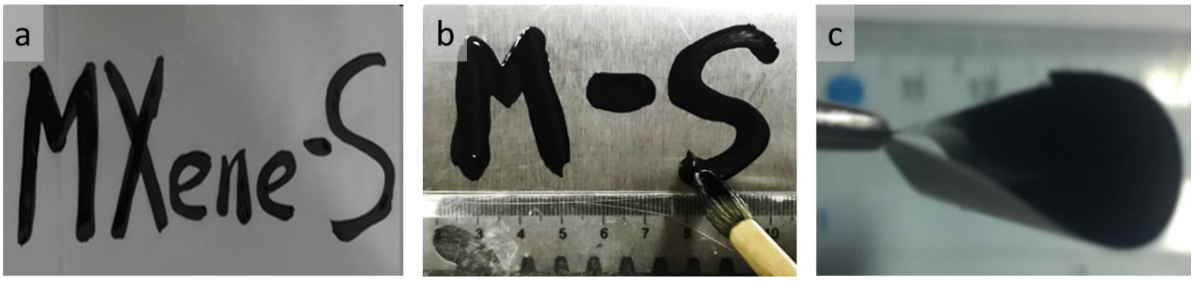

Figure S4. S@$Ti_3C_2T_x$ ink painted on (a) paper and (b) stainless steel. (c) vacuum-filtrated flexible, freestanding S@$Ti_3C_2T_x$ film.

The as-produced S@$Ti_3C_2T_x$ aqueous ink can be painted on arbitrary substrates such as paper (a) and stainless steel (b). Importantly, this ink can be slurry casted on the Al foil using an industry-compatible technique (doctor-blade method) without the need of any conductive agents or polymeric binder. The MXene nanosheets act as the conductive binder and provide both an advanced electron transport network and mechanical stability. In addition, the S@$Ti_3C_2T_x$ aqueous ink can be vacuum-filtrated to obtain the freestanding, flexible S@$Ti_3C_2T_x$ electrode, holding great promise for robust Li-S batteries.

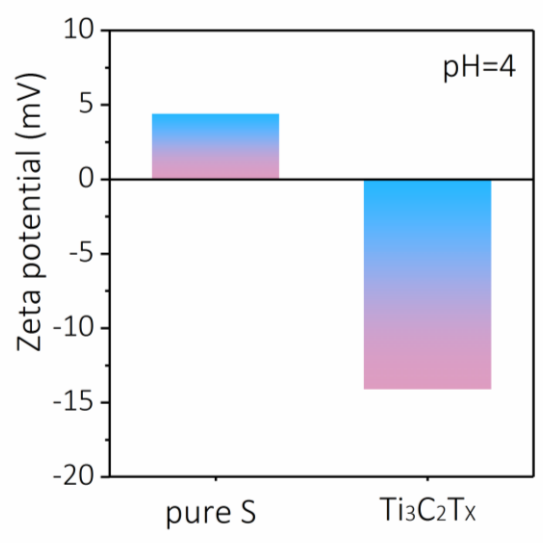

Figure S5. Zeta potential value of $Ti_3C_2T_x$ and *in-situ* formed S NPs.

The surface charges on the $Ti_3C_2T_x$ and S NPs are opposite, therefore, the nucleated S NPs are electrostatically absorbed on the $Ti_3C_2T_x$ surface so that the surface charges are screened.

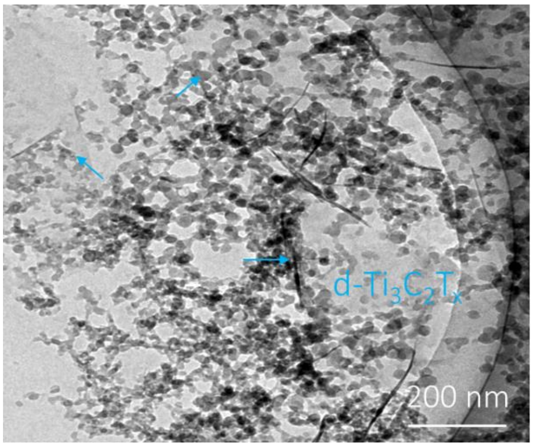

Figure S6. TEM image of S@$Ti_3C_2T_x$ aqueous ink showing that the sulfur NPs are homogeneously anchored on the $Ti_3C_2T_x$ nanosheets even after sonication, suggesting good interaction between the nanosheets and S NPs.

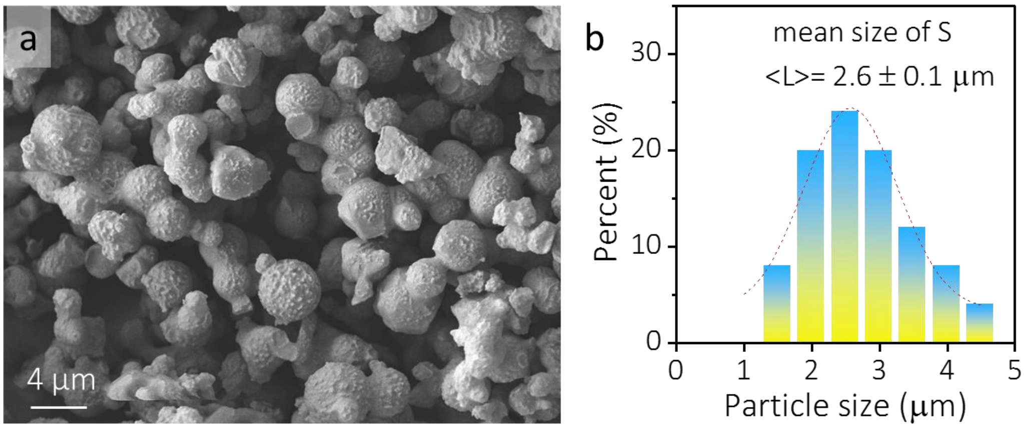

Figure S7. (a) SEM image and (b) particle size distribution of *in-situ* formed S without the addition of $Ti_3C_2T_x$, showing dimensions of ~µm.

Without the guiding/limiting effect from the $Ti_3C_2T_x$, pure S particles are no longer nanoscale, instead, the seeds agglomerated and formed microsized S with mean particle size ~2.6±0.1 µm. This controlled sample further highlighted the importance of MXene in uniformly guiding the deposition of S NPs.

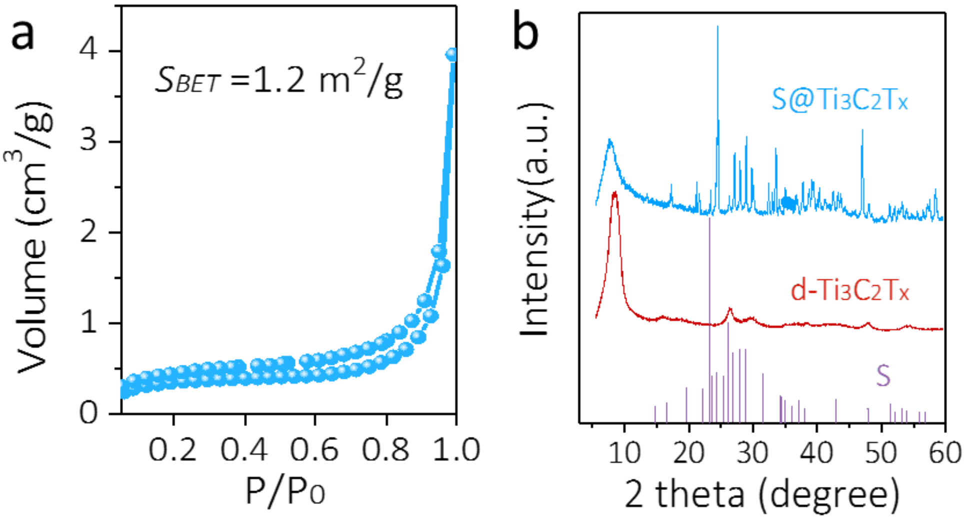

Figure S8. (a) N_2 isotherm and (b) XRD pattern of 70% S@$Ti_3C_2T_x$. For comparison, pure S and d-$Ti_3C_2T_x$ were also included. The S@$Ti_3C_2T_x$ composite possesses a compacted morphology thus leading to quite a low surface area.

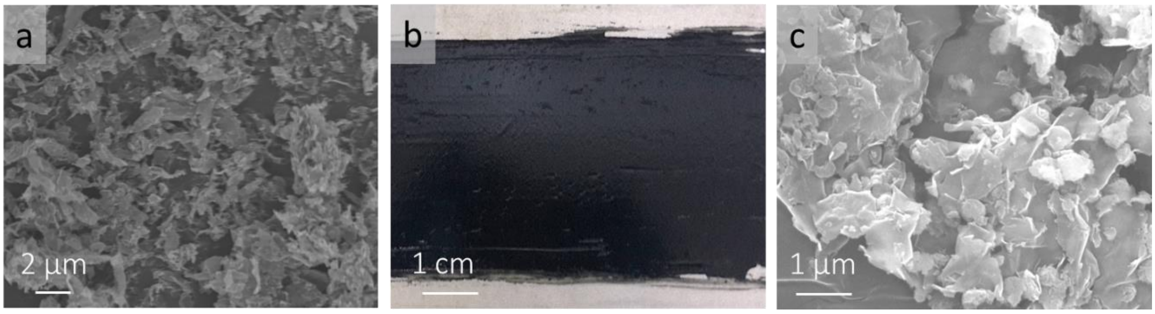

Figure S9. SEM image of (a) freeze-dried $Ti_3C_2T_x$ powder. (b) Optical image of $Ti_3C_2T_x$–S film made by casting the mixture (70% S + 20% $Ti_3C_2T_x$+ 10% polyvinylidene difluoride, PVDF) onto Al foil, showing non-uniformity over large areas. (c) SEM image of the $Ti_3C_2T_x$–S mixture electrode.

To show the synergistic effect between S NPs and d-$Ti_3C_2T_x$, the reference sample (conventional composite electrode) was prepared by physically mixing the pure S particles with freeze-dried $Ti_3C_2T_x$ and PVDF in a weight ratio of 7:2:1. The solvent was N-Methyl-2-pyrrolidone.

As shown in Figure S9(b), appreciable non-uniformity is observed in the slurry-casted film. The SEM image in (c) showcases the phase separation; the S particles detach from the flexible $Ti_3C_2T_x$ nanosheets. Such a nanostructure would impact the electron transport kinetics and as a result the electrochemical performance.

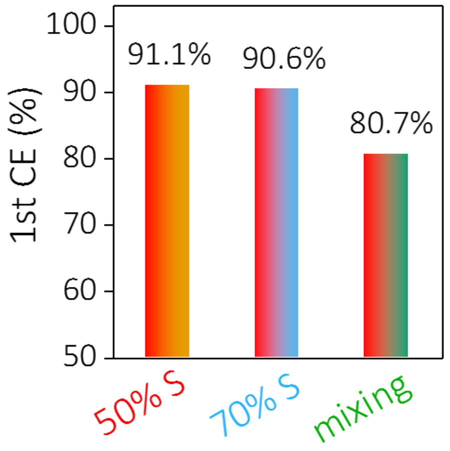

Figure S10. 1st cycle Coulombic efficiency of different composite electrodes

Compared to the $Ti_3C_2T_x$–S mixture electrode, the S@$Ti_3C_2T_x$ films showcase much higher 1st Coulombic efficiency (CB), reaching 90.6~91.1%. This means that much more reversible reactions have occurred at the liquid-solid interface of S@$Ti_3C_2T_x$ than those of the mixture electrode. The difference can be attributed to the charge-transfer resistance of these electrodes, as will be explained below.

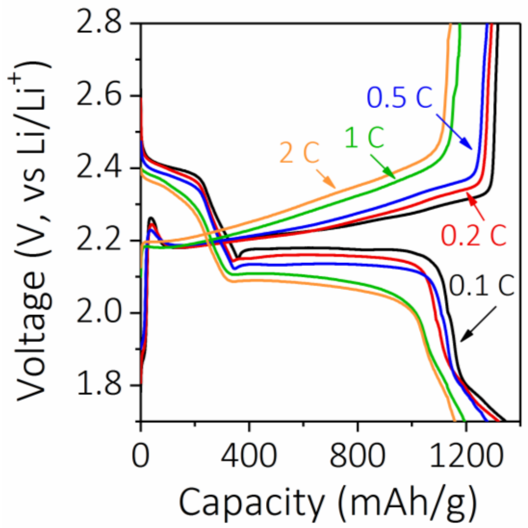

Figure S11. GCD of 50% S@$Ti_3C_2T_x$ at different C-rates.

The flexible electrode loaded with 50% S demonstrates high capacities and excellent rate capability. For example, when the C-rate increases 20 fold, the capacity slightly reduces from 1346 mAh/g to 1160 mAh/g. The Coulombic efficiency (~100%) coupled with a small polarization potential difference co-suggest that highly reversible reactions have been realized with the assistance of the $Ti_3C_2T_x$ conductive binder.

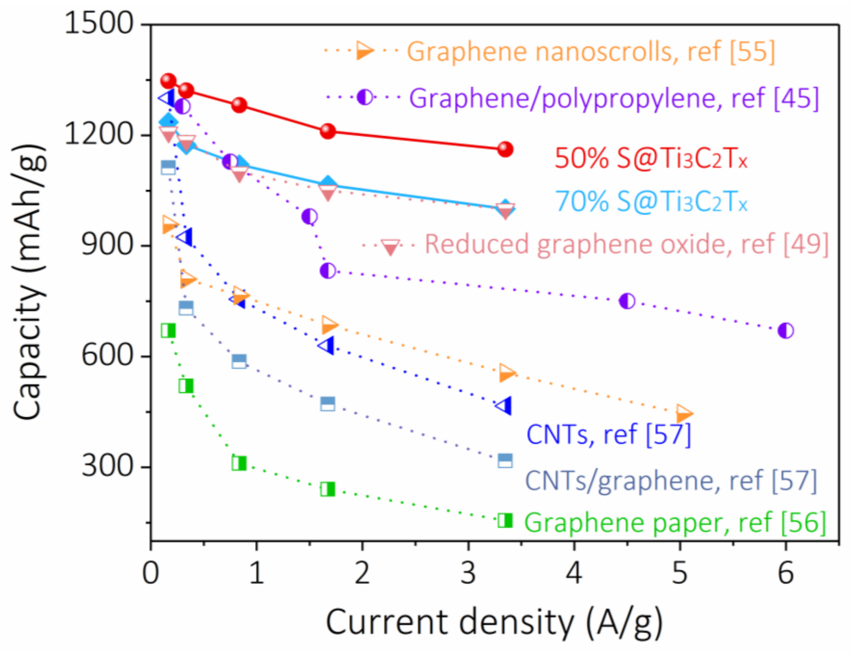

Figure S12. Comparison of the capacities in this work to those of various S-containing electrodes. Note, the references can be found in the manuscript.

While most non-polar mediators demonstrate quick capacity decay, such as graphene/polypropylene [45], graphene nanoscrolls [55], graphene paper [56], CNT or CNT/graphene [57], etc., the polar hosts (for instance, reduced graphene oxide [49]) on the other hand, showcase much better capacities at various C-rates. Among these S hosts, our 50% S@$Ti_3C_2T_x$ exhibits the highest capacities coupled with an excellent rate-handling property. In addition, the 70% S@$Ti_3C_2T_x$ displays a similar rate performance with lower capacities than the 50% S composite. Despite this, it still outperforms the capacities of 60% S/rGO [49], demonstrating the efficiency of the conductive $Ti_3C_2T_x$ mediator in maximizing S utilization.

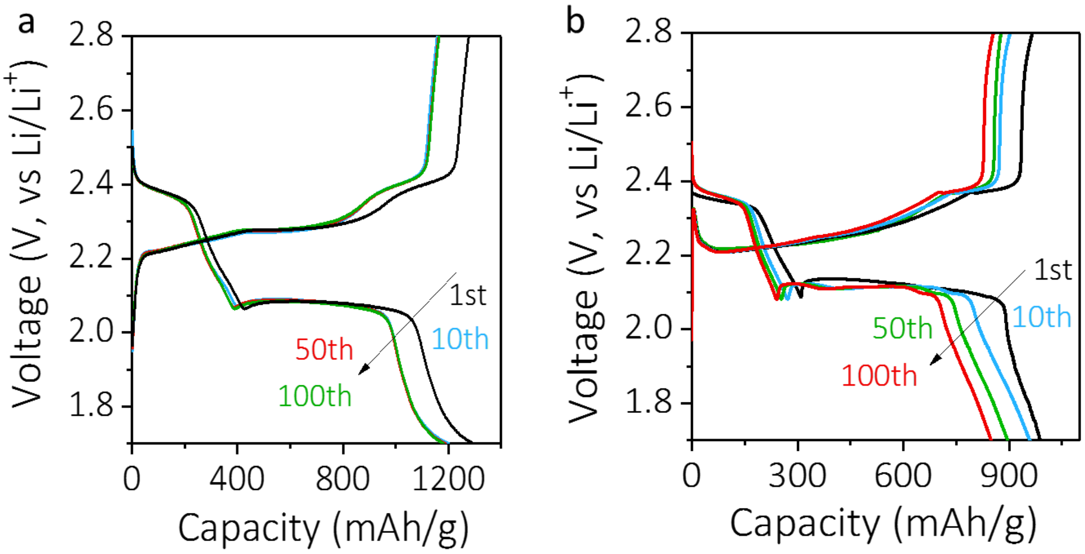

Figure S13. GCD curves of (a) 50% S and (b) 70% S@$Ti_3C_2T_x$ electrodes at different cycles.

Due to the unique role of $Ti_3C_2T_x$ in immobilizing the polysulfides (Li_2S_x), the S@$Ti_3C_2T_x$ films demonstrate excellent cycling performance. For instance, after a few cycles for stabilization, the 50% S@$Ti_3C_2T_x$ shows a stable capacity ~1200 mAh/g even after 100 cycles. On the other hand, due to the higher S loading, the 70% S@$Ti_3C_2T_x$ electrode displays an inferior lifetime performance. However, the polarization potential difference is almost constant upon cycling, suggesting that efficient charge-transfer kinetics have been maintained. The capacity decay is due to the loss of S in the form of an unavoidable Li_2S_x shuttle effect.

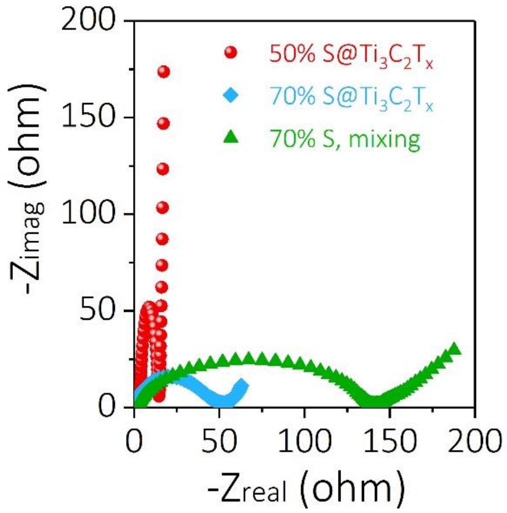

Figure S14. EIS Nyquist plots of several electrodes.

The above Nyquist plots of several electrodes clearly indicate the advantages of *in-situ* solution growth of S onto the conductive $Ti_3C_2T_x$ mediator. Due to the phase separation in the $Ti_3C_2T_x$–S mixture electrode, the charge-transfer kinetics at the liquid-solid interface are suppressed, especially during repeated electrochemical cycling. On the other hand, employing the $Ti_3C_2T_x$ mediator can effectively solve this issue, as seen in the much reduced semicircle, which represents the charge-transfer resistance (R_{ct}). When S loading is decreased, the R_{ct} decreases as expected.

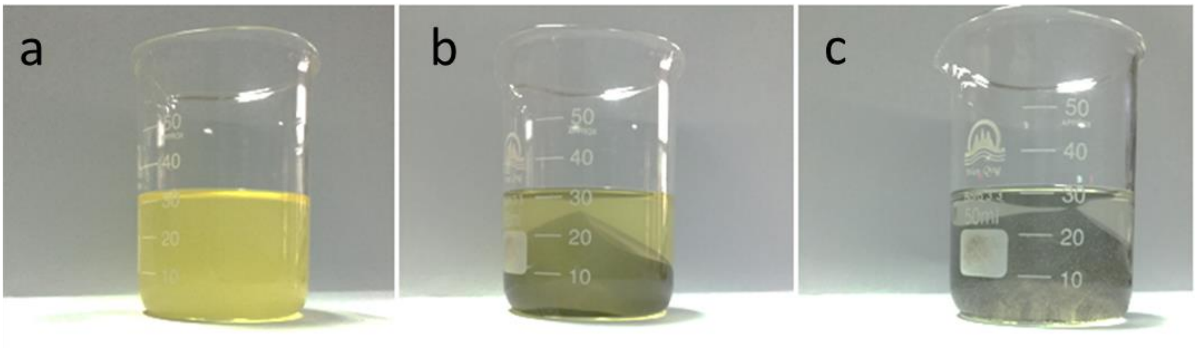

Figure S15. Photographs of (a) Li_2S_4 solution (artificial polysulfide solution); solution after (b) 20 min and (c) 1 hour interaction with a freestanding $Ti_3C_2T_x$ film.

To show the capability of $Ti_3C_2T_x$ in immobilizing the polysulfides, we used Li_2S_4 to represent the soluble Li_2S_x and immersed the freestanding $Ti_3C_2T_x$ into the solution. The bright yellow colour of Li_2S_4 quickly faded after 1 h interaction, as shown in (c), suggesting Li_2S_4 has been chemisorbed by the polar $Ti_3C_2T_x$ mediator. This confirms that $Ti_3C_2T_x$ possess abundant sites for the efficient chemisorption of the polysulfides.

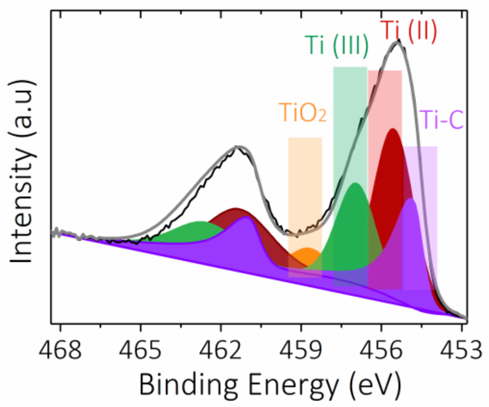

Figure S16. Ti 2p core level XPS spectrum of the pristine $Ti_3C_2T_x$, showing a similar spectrum to the reported data.[4,7]

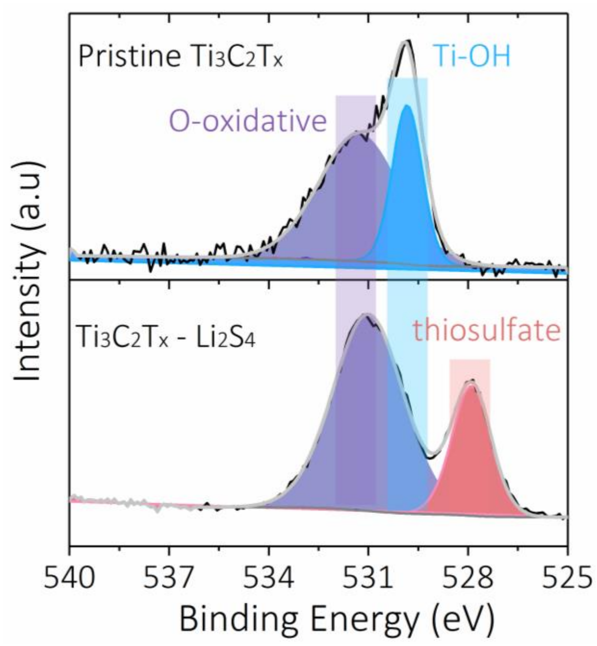

Figure S17. O1s core level XPS spectrum of the pristine $Ti_3C_2T_x$ and the $Ti_3C_2T_x$ electrode after interacting with Li_2S_4 solution. After immersing the $Ti_3C_2T_x$ film in the Li_2S_4solution for 1 h, the Ti-OH peak diminished, instead, a peak corresponding to thiosulfate appeared. This means that the –OH terminal groups reacted with Li_2S_4 and converted them into thiosulfate.

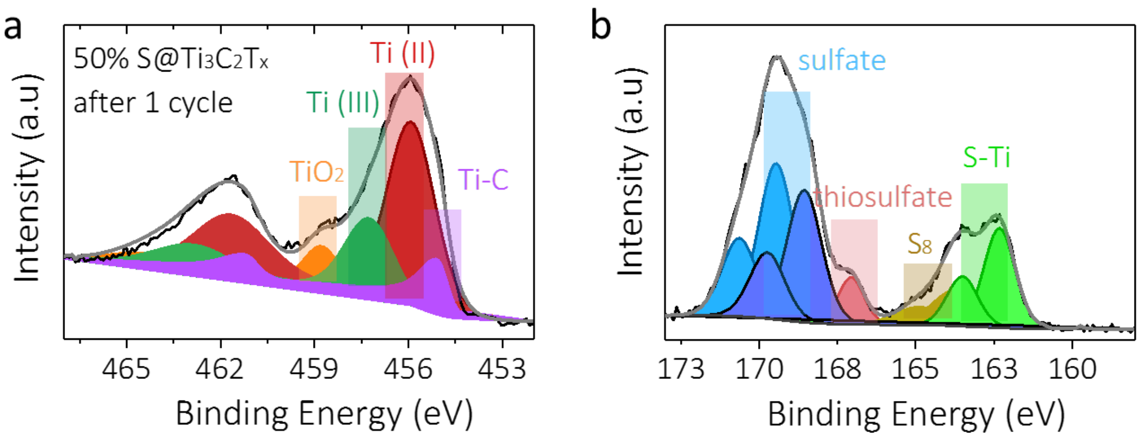

Figure S18. (a) Ti 2p and (b) S 2p core level XPS spectra of the 50% S@$Ti_3C_2T_x$ cathode after 1 cycle

The XPS spectra of the 50% S@$Ti_3C_2T_x$ cathode are quite similar to that of the 70% S@$Ti_3C_2T_x$ one. It's important to note that the Ti-S peak overlaps with that of Ti-C peak, rendering it hard to distinguish whether or not the Ti-S bond is formed from the Ti 2p spectrum alone. In the S 2p spectrum, a predominant sulfate peak is observed similar to that in the 70% S@$Ti_3C_2T_x$. In addition, a peak corresponding to the S-Ti bond is observed. We believe the sulfate complex layer acts as a protective barrier which retards the polysulfides shuttle. The sulfates layer coupled with the S-Ti bond result in an excellent lifetime for the S@$Ti_3C_2T_x$ based Li-S batteries.

References

[1] T. Zhang, L. Pan, H. Tang, F. Du, Y. Guo, T. Qiu, J. Yang, *J. Alloys Compd.* **2017**, *695*, 818.

[2] O. Mashtalir, M. Naguib, V. N. Mochalin, Y. Dall'Agnese, M. Heon, M. W. Barsoum, Y. Gogotsi, *Nat. Commun.* **2013**, *4*, 1716.

[3] M. Ghidiu, M. R. Lukatskaya, M.-Q. Zhao, Y. Gogotsi, M. W. Barsoum, *Nature* **2014**, *516*, 78.

[4] A. Lipatov, M. Alhabeb, M. R. Lukatskaya, A. Boson, Y. Gogotsi, A. Sinitskii, *Adv. Electron. Mater.* **2016**, *2*, 1600255.

[5] X. Liang, Y. Rangom, C. Y. Kwok, Q. Pang, L. F. Nazar, *Adv. Mater.* **2017**, *29*, 1603040.

[6] C. J. Zhang, B. Anasori, A. Seral-Ascaso, S.-H. Park, N. McEvoy, A. Shmeliov, G. S. Duesberg, J. N. Coleman, Y. Gogotsi, V. Nicolosi, *Adv. Mater.* **2017**, *29*, 1702678.

[7] C. J. Zhang, S. Pinilla, N. McEvoy, C. P. Cullen, B. Anasori, E. Long, S.-H. Park, A. Seral-Ascaso, A. Shmeliov, D. Krishnan, C. Morant, X. Liu, G. S. Duesberg, Y. Gogotsi, V. Nicolosi, *Chem. Mater.* **2017**, *29*, 4848.
